# Phagocytosis of Apoptotic Cells in Resolution of Inflammation

**DOI:** 10.3389/fimmu.2020.00553

**Published:** 2020-03-31

**Authors:** Ioannis Kourtzelis, George Hajishengallis, Triantafyllos Chavakis

**Affiliations:** ^1^Institute for Clinical Chemistry and Laboratory Medicine, University Hospital and Faculty of Medicine, Technische Universität Dresden, Dresden, Germany; ^2^Hull York Medical School, York Biomedical Research Institute, University of York, York, United Kingdom; ^3^Laboratory of Innate Immunity and Inflammation, Penn Dental Medicine, Department of Basic and Translational Sciences, University of Pennsylvania, Philadelphia, PA, United States; ^4^Centre for Cardiovascular Science, Queen’s Medical Research Institute, University of Edinburgh, Edinburgh, United Kingdom

**Keywords:** phagocytosis, efferocytosis, DEL-1, immunometabolism, inflammation resolution, integrins

## Abstract

Efficient inflammation resolution is important not only for the termination of the inflammatory response but also for the restoration of tissue integrity. An integral process to resolution of inflammation is the phagocytosis of dying cells by macrophages, known as efferocytosis. This function is mediated by a complex and well-orchestrated network of interactions amongst specialized phagocytic receptors, bridging molecules, as well as “find-me” and “eat-me” signals. Efferocytosis serves not only as a waste disposal mechanism (clearance of the apoptotic cells) but also promotes a pro-resolving phenotype in efferocytic macrophages and thereby termination of inflammation. Alterations in cellular metabolism are critical for shaping the phenotype and function of efferocytic macrophages, thus, representing an important determinant of macrophage plasticity. Impaired efferocytosis can result in inflammation-associated pathologies or autoimmunity. The present mini review summarizes current knowledge regarding the mechanisms regulating macrophage efferocytosis during clearance of inflammation.

## Introduction

Specific recognition and engulfment of “foreign” material or pathogens by host cells, designated as phagocytosis, is an essential process modulating the immune response and tissue homeostasis (1, 2). Besides phagocytosis of opsonized pathogens by phagocytes during an infection, host cells undergoing apoptosis are also cleared by macrophages; the specific phagocytosis of dying cells by macrophages is designated efferocytosis (3). The specific pathways for phagocytosis of different types of cargo point to the versatility of the phagocytic machinery (1, 2).

**FIGURE 1:**
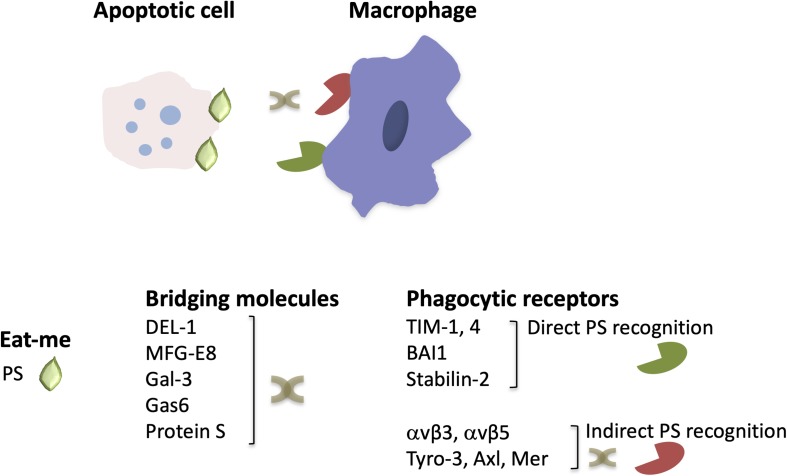
The structure of engulfment synapse. During efferocytosis, the clearance of apoptotic cells by macrophages is orchestrated by the recognition of the major “eat-me” signal PS, either directly by PS receptors or via bridging molecules that mediate binding of PS to phagocytic receptors. Depicted are a few examples of phagocytic receptors and bridging molecules that are described in the text (see text for primary references).

A central player in sterile inflammation or inflammation associated with infection are neutrophils recruited to the inflamed site (4, 5). Recruited neutrophils phagocytose and kill pathogens, produce reactive oxygen species (ROS) and pro-inflammatory factors, such as cytokines, and can either release neutrophil extracellular traps (NETs) or undergo apoptosis (4–6). Efferocytosis is therefore of great importance in the regulation of neutrophilic inflammation (3, 7–10). Effective removal of dying neutrophils promotes not only inflammation resolution but also contributes to restoration of tissue and organ homeostasis (3). A complex network of interactions between receptors mediating phagocytosis, bridging molecules, “find-me” and “eat-me” signals, such as phosphatidylserine (PS), which is presented on the outer part of the membrane of cells undergoing apoptosis, contributes to the formation of phagocytic synapse (Figure 1) and the operation of the efferocytic machinery (7, 8, 11). As resolution of inflammation occurs, efferocytic macrophages acquire a resolving phenotype producing factors that dampen inflammation and promote restoration of tissue integrity, such as IL-10 or transforming growth factor β (TGFβ) (3), as as well as specialized pro-resolving lipid mediators (SPM), such as resolvins, lipoxins, and maresins (9, 12). SPM synthesis can further promote efferocytosis, thereby further potentiating inflammation resolution (9, 12). Defective removal of apoptotic cells resulting from impaired efferocytosis can lead to chronicity of inflammation and development of inflammatory disorders, such as atherosclerosis and autoimmune diseases (9, 13–15).

**FIGURE 2:**
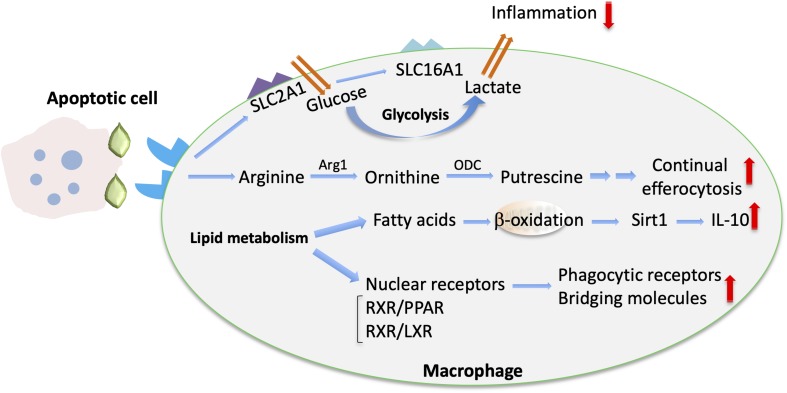
Metabolic cues implicated in macrophage efferocytosis. Upon efferocytosis, increased glucose uptake via upregulated SLC2A1 and enhanced glycolysis are linked with enhanced lactate release via SLC16A (102). The metabolism of arginine and ornithine to putrescine is involved in promoting continual efferocytosis (105). In addition, enhanced lipid metabolism upon efferocytosis is associated with fatty acid oxidation and Sirtuin 1 (Sirt1)-dependent upregulation of IL-10 (18). Moreover, activation of lipid transcription factors (e.g., the LXR/RXR or PPAR/RXR heterodimers) promotes the upregulation of bridging molecules and phagocytic receptors and the resolving macrophage phenotype (107).

Emerging evidence suggests that macrophage function is regulated by alterations in their cellular metabolism in response to environmental cues within the inflammatory milieu (16). For instance, specific metabolic components may promote or suppress inflammatory responses in macrophages (16). Importantly, efferocytosis also promotes immunometabolic reprograming in macrophages (17) (Figure 2). For example, digestion of the engulfed apoptotic cargo through phagolysosomal activity results in a load of lipid components derived from the apoptotic cell membranes that is linked to enhanced fatty acid oxidation and regulates macrophage function (17–19). The mechanisms underlying apoptotic cell removal by macrophages as well as the immunometabolic alterations in efferocytic macrophages during inflammation resolution is the focus of the present review.

## Molecular Cross-Talk Between Apoptotic Cells and Macrophages During Efferocytosis

The engulfment of dying cells by efferocytic macrophages requires the recognition of the former by the latter and the formation of the engulfment synapse, which is regulated by a network of “find-me,” “eat-me” and bridging molecules, “don’t eat-me” signals and specialized phagocytic receptors (11) (Figure 1). Neutrophils undergoing apoptosis during an inflammatory response, secrete molecules serving as “find-me” signals that can attract phagocytes to eliminate apoptotic cell corpses (20, 21). These include the nucleotides adenosine triphosphate (ATP) and uridine triphosphate (UTP), which are recognized by the macrophage purinergic receptor P2Y2 (22), or the lipids lysophosphatidylcholine (LPC) (23) and sphingosine-1-phosphate (24), which bind to macrophage G-protein-coupled receptors G2A and S1P1-5, respectively. Furthermore, recognition of dying cells by macrophages may be facilitated by the interaction of intercellular adhesion molecule 3 (ICAM3 or CD50) on the former with CD14 on macrophages (25) as well as by the thrombospondin (TSP1)–CD36 interaction (26). Moreover, the specific recognition of apoptotic cells is ensured by the presence of “eat-me” signals. PS is the most well characterized “eat-me” signal (Figure 1). During apoptosis, this phospholipid is found on the outer part of the membrane and binds directly or indirectly, via bridging molecules (opsonins), to phagocytic receptors (27, 28). Calreticulin (Crt) is a membrane-associated protein that functions as an “eat-me” signal’ on the surface of dying cells and is recognized by the LDL-receptor-related protein 1 (LRP1 or CD91) on phagocytes (29). The long pentraxin PTX3 may also act as an “eat-me” signal to facilitate the capture of dying neutrophils by macrophages (30).

As alluded to above, bridging molecules are often key to efficient interactions between apoptotic neutrophils and macrophages (11). Milk fat globule-EGF factor 8 protein (MFG-E8 or lactadherin) promotes efferocytosis by binding to PS on apoptotic cells and to macrophage phagocytic integrin receptors αvβ3 and αvβ5 (31, 32). MFG-E8 shares homology with developmental endothelial locus-1 (DEL-1). Besides the established anti-inflammatory role of DEL-1 as inhibitor of β2 integrin-dependent leukocyte recruitment and IL-17-mediated inflammation (8, 33–38) and its role as a regulator of bone marrow myelopoiesis (39, 40), secreted DEL-1 promotes engulfment of apoptotic cells and inflammation resolution (41). In particular, DEL-1 functions as a molecular bridge that binds concomitantly to PS on the apoptotic neutrophil surface via its C-terminal discoidin I-like domains and to αvβ3 integrin [also known as vitronectin receptor (42)] on macrophages via its N-terminal RGD motif within the second EGF-like domain (41, 43). Importantly, the compartmentalized localization of DEL-1 differentially regulates the inflammatory response. Specifically, the endothelial cell-derived molecule promotes anti-inflammatory activity by blocking β2 integrin-dependent leukocyte recruitment, whereas DEL-1 derived from macrophages promotes efferocytosis-dependent resolution of inflammation (33, 41) (Figure 1). Other molecules that act as a molecular bridge to facilitate interactions between apoptotic cargo and phagocytes include annexin A1 or lipocortin-1 (44), β2-glycoprotein-I (β2-GPI) (45), and galectin-3 (Gal-3) (46). Moreover, the bridging molecules Growth arrest-specific factor 6 (Gas6) and protein S have been implicated in PS-mediated apoptotic cell clearance (47, 48) via interacting with the receptor tyrosine kinases Tyro-3, Axl and Mer (TAM) (49–52) (Figure 1).

Phagocytic receptors on macrophages involved in the regulation of efferocytosis include also the PS receptors of the T-cell membrane protein (Tim) family, such as TIM-1 and TIM-4 (53, 54). The brain angiogenesis inhibitor 1 (BAI1) (55) and stabilin-2 (56) also serve as PS receptors (Figure 1). Additionally, CD14, the scavenger receptor CD36, and the integrin CD11b/CD18 (αMβ2) (besides the integrins αvβ5 and αvβ3 that were mentioned above) are implicated as efferocytosis receptors (8, 11, 26, 42, 57, 58).

The presence of “don’t eat-me” signals further adds to the complexity of the regulation of apoptotic cell clearance. Specifically, surface expression of CD47 (also named integrin-associated protein) prevents phagocytosis by macrophages (59, 60). Binding of CD47 to the macrophage signal regulatory protein alpha (SIRPα) modulates rearrangement of actin cytoskeleton, thereby downregulating phagocytosis (59, 60). However, apoptotic cells have decreased levels of CD47 that allows their clearance by macrophages (59–61). Platelet and endothelial cell adhesion molecule 1 (PECAM-1, CD31) also exerts a “don’t eat-me” function. In this regard, homotypic CD31 interaction between non-apoptotic neutrophils and macrophages may prevent phagocytic clearance (62). Furthermore, CD24 (63) and the complement receptor CD46 (64) have been described as repulsive signals that interfere with efferocytosis. Decreased presence or alterations in the distribution of “don’t eat-me” signals have been associated with enhanced efferocytic activity (65, 66).

Efficient efferocytosis is critical for shaping the pro-resolving phenotype in macrophages that includes production of immunomodulators, which in turn further enhance resolution of inflammation (67–71). For instance, production of TGFβ by efferocytic macrophages is a major orchestrator of inflammation resolution. Indeed, upregulation of TGFβ owing to efferocytosis promotes downregulation of the pro-inflammatory mediators TNF, IL-1β and IL-8. Consistently, antibody-mediated inhibition of TGFβ restored expression of inflammatory mediators. Along the same line, administration of apoptotic cells *in vivo* models of inflammation triggers resolution of inflammation in a manner dependent on TGFβ upregulation (72, 73). Additionally, interleukin 13 derived from regulatory T cells acts on macrophages and promotes production of IL-10, which in turn enhances efferocytosis via activation of Rac1 GTPase and thereby inflammation resolution in atherosclerosis (74). Besides the upregulation of immune-modulating factors, such as TGFβ or IL-10, the direct inhibition of pro-inflammatory cytokines further contributes to inflammation resolution. As an exemplar, formation of NETs that aggregate at the inflamed site leads to protease–dependent degradation of inflammatory cytokines and chemokines, thereby promoting resolution of acute neutrophilic inflammation (75).

The phenotype of efferocytic macrophages is additionally controlled by the enzyme 12/15 lipoxygenase (12/15-LO) that oxidizes polyunsaturated fatty acids and generates bioactive lipid metabolites leading to the biosynthesis of pro-resolving lipid mediators (9, 76). Specifically, apoptotic cell engulfment can be performed by resident or monocyte-derived resolution phase macrophages expressing 12/15-LO (77–80). Apoptotic cell engulfment further promotes the expression of this enzyme (81). Moreover, 12/15-LO has been implicated to function in preventing induction of autoimmunity (77).

Plasminogen and its cleavage product plasmin not only regulate the initiation but also the resolution phase of inflammation. Treatment of mice with plasminogen/plasmin resulted in recruitment of pro-resolving macrophages and in upregulation of TGFβ. Administration of plasminogen/plasmin at the peak of inflammation was associated with increased neutrophil apoptosis and efferocytosis; the pro-resolving effect of plasminogen was mediated by annexin A1 (82). In accordance, impaired efferocytosis accompanied by decreased levels of annexin A1 was observed in mice deficient in plasminogen or its receptor (83). Besides being involved in pro-resolving actions of plasminogen, annexin A1 plays a broader role in inflammation resolution (84, 85). Annexin A1 levels are increased in the resolution phase of monosodium urate crystal–induced arthritis, a model of gout. Pharmacologic or genetic inactivation of annexin A1 resulted in insufficient resolution of gout-related inflammation in mice (86). In addition, treatment of mice with annexin A1 resulted in upregulation of IL-10 and downregulation of proinflammatory mediators, while, consistently, inhibition of annexin A1 abrogated inflammation resolution induced by glucocorticoids (87, 88).

Moreover, IFN-β from macrophages was recently identified as a factor promoting resolution of inflammation. IFN-β levels were higher in the resolution phase of pneumonia and peritoneal inflammation. Activation of IFN-β signaling via STAT3 enhances apoptosis of neutrophils and their subsequent efferocytic clearance, resulting in a pro-resolving reprograming of macrophages (79).

## Metabolic Modulation of Macrophage Function in the Context of Efferocytosis

The impact of cellular metabolism on macrophage function and plasticity has gained much attention recently (16, 89–91). Metabolic pathways, such as glycolysis, tricarboxylic acid (TCA) cycle, pentose phosphate pathway and fatty acid oxidation, regulate macrophage phenotype in the context of inflammatory responses (91). For instance, increased glycolytic flux has been linked to pro-inflammatory M1-like activation of macrophages, whereas oxidative phosphorylation is associated with anti-inflammatory macrophage polarization (92).

Moreover, macrophage tissue specificity may be associated with differential metabolic activity. For example, resident peritoneal macrophage survival depends on the transcription factor GATA6 that is regulated by the vitamin A metabolite retinoic acid (93, 94), while the nuclear receptor liver × receptor (LXR) alpha that is activated by lipids regulates differentiation of marginal zone splenic macrophages (95). It is now established that tissue-specific resident macrophages have distinct transcriptomic profiles and phenotypes depending on the particular microenvironment (96, 97). Importantly, in this regard, the manner by which efferocytosis is regulated in resident macrophages may be dictated by the tissue microenvironment. Indeed, parabiosis-based experiments have revealed substantial heterogeneity in the utilization of bridging molecules, efferocytic receptors and transcription factors by macrophages from different tissues. For instance, the mannose receptor CD206 is upregulated in phagocytic macrophages in bone marrow and intestine but not in the spleen (98). Moreover, the profile of upregulated anti-inflammatory mediators by efferocytic macrophages is tissue-specific (98).

Regulation of macrophage metabolic activity in the context of efferocytosis (Figure 2) is of major importance for the outcome of inflammation resolution and tissue repair (99). Following engulfment, macrophages degrade the apoptotic material through phagolysosomal activity (100, 101), resulting in substantial metabolic load and influence on macrophage metabolism (17).

Aerobic glycolysis was recently implicated in regulation of efferocytosis and shaping an anti-inflammatory environment by efferocytic macrophages (102). Specifically, transcriptomic analysis of macrophages engaging in active phagocytosis of apoptotic cells revealed an upregulation of several members of the solute carrier (SLC) membrane transport protein family, including the glucose transporter protein type 1 (GLUT1; encoded by the gene *Slc2a1*) and monocarboxylate transporter 1 (encoded by *Slc16a1*) promoting lactate release. Enhanced glycolysis in efferocytic macrophages promoted actin polymerization and continued uptake of apoptotic cells. On the other hand, lactate from efferocytic macrophages contributed to establishment of an anti-inflammatory phenotype of the surrounding tissue (102) (Figure 2). Consistently, knockdown of *Slc16a1* in the setting of efferocytosis resulted in reduced mRNA expression of factors linked to the resolving macrophage phenotype and resolution of inflammation, such as *Tgfb1* and *Il10* (102). Furthermore, specific deletion of GLUT1 in myeloid cells was associated with defective phagocytic ability of macrophages and with development of unstable lesions in a model of atherosclerosis (103). Moreover, deficiency of the glycolytic enzyme 6-phosphofructose-2-kinase and fructose-2,6-bisphosphatase (PFKFB3) in macrophages led to reduced efferocytosis capacity, thus further supporting a role for glycolysis in apoptotic cell clearance (104).

Metabolites derived from engulfed apoptotic cells serve to fine-tune the process of efferocytosis and resolution of inflammation. Apoptotic cells are a source of the amino acids arginine and ornithine that are metabolized in macrophages to putrescine. This metabolic process enables continual rounds of efferocytosis (Figure 2). Putrescine enhances, via the RNA-binding protein HuR, the mRNA stabilization of the GTP-exchange factor Dbl. Dbl in turn activates the GTPase Rac1, resulting in changes in the actin cytoskeleton that facilitate further apoptotic cell engulfment. Exogenous administration of putrescine increases inflammation resolution in the context of atherosclerosis. Consistently, deficiency in myeloid cells of either the enzyme arginase 1 (Arg1), which converts arginine to ornithine, or the enzyme ornithine decarboxylase (ODC), which mediates the decarboxylation of ornithine to putrescine (Figure 2), is associated with efferocytic dysfunction and defective atherosclerosis resolution (105).

Several lines of evidence support also an important role of mitochondrial metabolism on the modulation of efferocytosis and efferocytosis-dependent resolution of inflammation. Metabolomic analysis of macrophages that have engulfed apoptotic material revealed metabolic reprograming of efferocytic macrophages toward fatty acid oxidation. Specifically, upregulation of pro-resolving IL-10 in efferocytic macrophages was mediated by mitochondrial beta-oxidation and induction of sirtuin1 signaling (18) (Figure 2). The interplay between mitochondrial activity and effective efferocytosis was also illustrated by analyzing the function of mitochondrial uncoupling protein 2 (Ucp2). Besides its function in uncoupling oxidative phosphorylation from synthesis of ATP, Ucp2 can promote efferocytosis by reducing the mitochondrial membrane potential of macrophages. In the same vein, deficiency of Ucp2 resulted in defective apoptotic cell removal and was associated with development of atherosclerosis, whereas overexpression of Ucp2 enhanced efferocytosis (19). Additionally, dynamic alterations in mitochondrial physiology may dictate the outcome of efferocytosis. In particular, a major component of mitochondrial homeostasis, mitochondrial fission, mediated by the function of the GTPase dynamin-related protein 1 (Drp1), positively regulates continuous apoptotic cell removal by macrophages. Accordingly, the absence of Drp1 was associated with higher atherosclerosis development in low-density lipoprotein receptor 1 (*Ldlr1)* deficient mice (106).

Lipids deriving from the apoptotic cargo are abundant post-engulfment digestion products (17); lipid metabolism induces activation of the nuclear receptors peroxisome proliferator-activated receptor (PPAR) gamma and delta, LXR alpha and beta and retinoid × receptor (RXR) alpha and beta (107) in macrophages (Figure 2). Activation of these transcription factors promotes upregulation of phagocytic receptors and bridging molecules and the resolving macrophage phenotype. For instance, TGFβ and IL-10, which both promote inflammation resolution, are upregulated in efferocytic macrophages in an LXR- and PPAR delta–dependent manner (108, 109). Importantly, deficiency of these nuclear receptors has been linked to defective efferocytosis and development of chronic inflammation or autoimmune manifestations (108, 109). Moreover, LXR activation is involved in DEL-1–dependent efferocytosis and macrophage reprograming to a proresolving phenotype (41). LXR signaling also regulates expression of transglutaminase 2 (Tgm2) (110), which acts as a co-receptor to αvβ3-integrin and promotes formation of engulfing portals (111).

Cholesterol metabolism has been also implicated in the modulation of efferocytosis. Treatment of macrophages with the cholesterol-lowering drug lovastatin, which inhibits the rate-limiting enzyme of cholesterol synthesis 3-hydroxyl-3-methylglutaryl coenzyme A (HMG-CoA) reductase, leads to increased apoptotic cell clearance (112). Administration of another HMG-CoA reductase inhibitor, simvastatin, enhances the efferocytosis-dependent amelioration of inflammation in the context of lung fibrosis (113). Along the same line, the ATP-binding cassette transporter (ABCA1), a protein that modulates cholesterol efflux, is upregulated during efferocytosis in a manner dependent on LXR signaling (114). Furthermore, decreased hydrolysis of cholesterol esters and impaired oxysterol production, due to blockade of the enzyme lysosomal acid lipase, negatively affect LXR pathway activation and apoptotic cell removal, resulting in chronic inflammation (115). These studies point to the intimate crosstalk between cholesterol metabolism and nuclear receptor signaling involved in efferocytosis.

## Concluding Remarks

Macrophage efferocytosis is a major player in resolution of inflammation. Efferocytosis not only paves the way toward the timely termination of the inflammatory response, but also promotes restoration of tissue homeostasis. In this context, alterations in macrophage cellular metabolism are important regulators of efferocytosis. At the same time, metabolic reprograming in efferocytic macrophages induced by digested apoptotic material substantially regulates the function and plasticity of efferocytic macrophages. Given the relevance of efferocytosis as a mechanism against chronic inflammatory disease, a better mechanistic understanding of the pathways that orchestrate the mutual interaction between clearance of dying cells and metabolic alterations in macrophages is required. This knowledge will provide a scaffold for designing therapeutic approaches to improve macrophage function in inflammation resolution and harness macrophage efferocytosis for the treatment of pathologies associated with chronic inflammation or autoimmunity.

## Author Contributions

IK, GH, and TC wrote the manuscript.

## Conflict of Interest

The authors declare that the research was conducted in the absence of any commercial or financial relationships that could be construed as a potential conflict of interest.
